# High-Throughput Design of Two-Dimensional Electron Gas Systems Based on Polar/Nonpolar Perovskite Oxide Heterostructures

**DOI:** 10.1038/srep34667

**Published:** 2016-10-06

**Authors:** Kesong Yang, Safdar Nazir, Maziar Behtash, Jianli Cheng

**Affiliations:** 1 Department of NanoEngineering, University of California, San Diego, 9500 Gilman Drive, Mail Code 0448, La Jolla, California 92093-0448, USA

## Abstract

The two-dimensional electron gas (2DEG) formed at the interface between two insulating oxides such as LaAlO_3_ and SrTiO_3_ (STO) is of fundamental and practical interest because of its novel interfacial conductivity and its promising applications in next-generation nanoelectronic devices. Here we show that a group of combinatorial descriptors that characterize the polar character, lattice mismatch, band gap, and the band alignment between the perovskite-oxide-based band insulators and the STO substrate, can be introduced to realize a high-throughput (HT) design of SrTiO_3_-based 2DEG systems from perovskite oxide quantum database. Equipped with these combinatorial descriptors, we have carried out a HT screening of all the polar perovskite compounds, uncovering 42 compounds of potential interests. Of these, Al-, Ga-, Sc-, and Ta-based compounds can form a 2DEG with STO, while In-based compounds exhibit a strain-induced strong polarization when deposited on STO substrate. In particular, the Ta-based compounds can form 2DEG with potentially high electron mobility at (TaO_2_)^+^/(SrO)^0^ interface. Our approach, by defining materials descriptors solely based on the bulk materials properties, and by relying on the perovskite-oriented quantum materials repository, opens new avenues for the discovery of perovskite-oxide-based functional interface materials in a HT fashion.

To overcome the quantum limitations of miniaturization of silicon-based electronic devices, one of the technical challenges for next-generation nanoelectronics is to achieve and control extremely high charge carrier densities and mobilities within structures at nanoscales^1^,^2^. In this respect, all-oxide electronics represent one of the most promising technologies for next-generation nanoelectronic devices beyond traditional silicon technology^3^,^4^,^5^, and producing two-dimensional electron gas (2DEG) at the interface of heterostructures (HS) is one of the most attractive technologies for nanoelectronics application^6^,^7^. The recent discovery of 2DEG at the polar/nonpolar (LaO)^1+^/(TiO_2_)^0^ interface between LaAlO_3_ (LAO) and SrTiO_3_ (STO) perovskite insulators opens a new avenue to prepare highly conductive oxides^8^,^9^, though its fundamental mechanism for the 2DEG formation is still on the debate. Several mechanisms such as polar catastrophe (that is considered as the primary reason)^8^,^9^,^10^, oxygen vacancy defects^11^,^12^,^13^,^14^, cation intermixing at the interface^9^,^10^,^15^ and even surface protonation^16^,^17^ have been proposed to explain the interfacial conductivity. In spite of the controversial formation mechanism of the 2DEG, its low room-temperature mobility is still a critical problem, and is very sensitive to the growth condition, in particular under varied oxygen partial pressures^8^,^9^,^10^,^11^,^12^,^13^,^18^,^19^.

To achieve potential applications of the 2DEG at the oxide interfaces in the high-performance nanoelectronic devices, in addition to fine-tuning its conductivity using various experimental techniques such as strain engineering^20^,^21^,^22^,^23^,^24^ and doping engineering^25^,^26^,^27^,^28^,^29^, one possible solution is to search for novel perovskite-based 2DEG systems with properties potentially superior to that of the LAO/STO system. However, the traditional process of experimental materials design in laboratory is a trial-and-error process and is bound by high time- and cost-consumption. The emerging high-throughput (HT) computational materials design approach provides an ideal solution for accelerating the materials discovery process^30^,^31^,^32^. Hence, to search for novel perovskite-based 2DEG systems with superior properties, one unique and efficient approach is to screen all the possible perovskite oxides and rapidly locate the target systems using the HT computational materials design approach.

In this work, by defining a group of combinatorial descriptors including the polar character, lattice mismatch, band gap, and band alignment between the polar perovskite oxides and the STO substrate, and by producing a perovskite-oxide-oriented quantum materials repository using the automatic framework AFLOW that is designed for HT materials discovery^33^,^34^, we have searched this quantum materials repository and rapidly extracted a total of 42 candidate perovskite oxides for further exploring the possibility of producing 2DEG at the interface.

## Results

The key to the success of HT materials screening is the development of accessible materials descriptors. The materials descriptors could be any identifiable materials characters and even a combination of several materials characters that are closely related to the desired materials properties. In terms of the perovskite-based 2DEG systems, the definition of the materials descriptors are built on the formation mechanism of the 2DEG, that is the polar catastrophe. In this work, we take the prototype LAO/STO system as an example, and extract a group of combinatorial descriptors for the HT discovery of the perovskite-based 2DEG systems based on the polar catastrophe mechanism, as detailed below.

According to the polar catastrophe mechanism, perovskite-based HS systems must satisfy several necessary conditions to form a 2DEG at the interface. First, the HS must be composed of polar and non-polar wide-band-gap perovskite oxides. Hence, the “polar/nonpolar” character will be used as the first descriptor to determine the substrate and the film. Specifically, this requires that the substrate is a nonpolar oxide (A^+2^B^+4^O_3_) such as STO and SrZrO_3_ while the film is a polar oxide (A^+1^B^+5^O_3_ or A^+3^B^+3^O_3_) such as LAO and LaGaO_3_. Herein, as a proof of concept, we choose the well-known nonpolar crystal STO as the reference substrate material. One reason is that the STO single crystal has a good lattice match with most perovskite materials, and is considered as an excellent substrate for epitaxial film growth. Consequently, after choosing the appropriate reference substrate material, next we will develop other descriptors for screening appropriate polar oxides as films. Nevertheless, it is worth mentioning that, from the perspective of the actual experimental film growth, the thickness of the polar perovskite film must be above a certain critical thickness^35^. For example, a minimum thickness of about 4 unit cells of LAO is necessary to form the 2DEG in the LAO/STO system^18^.

Second, the lattice constant of the deposited polar perovskite film should be close to that of the substrate, in this case STO. This is because a close lattice match between an epitaxially grown film and its substrate is beneficial to minimize defects and to improve the electron mobility^36^. Hence, the magnitude of the lattice mismatch between the polar perovskite and STO substrate will be used as the second descriptor to further screen the candidate systems. We define the lattice mismatch *f* as *f* = (*a*_*f*_ − *a*_*s*_)/*a*_*s*_, where *a*_*f*_ and *a*_*s*_ are the lattice constants of the unstrained film (polar perovskite) and the substrate (STO), respectively. This definition is widely used, although other similar definitions are also often adopted^37^. For example, it is noted that sometimes a denominator of *a*_*f*_ rather than *a*_*s*_ is adopted in defining the lattice mismatch, in which case the value of the lattice mismatch value is slightly different. A negative value of *f* indicates that the lattice constant of the deposited film is less than that of the substrate, suggesting that the film would suffer from a tensile strain. A positive value of *f* implies that the film would undergo a compressive strain. Therefore, 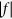 represents the degree of the lattice mismatch between the deposited film and substrate. By employing 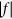 as the second descriptor, polar perovskite oxides that have a lattice mismatch 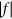 smaller than 8% with STO are further selected.

Third, to realize its practical implementation in the electronic devices, the 2DEG must be tightly confined in the interfacial region (in other words, insulating in the third dimension perpendicular to the interfacial plane) to reduce current leakage, which requires that the 2DEG must be formed at the interface between two insulators. It is known that there are three classes of insulators: conventional band insulators like SrTiO_3_^38^, topological insulators like Bi_2_Te_3_^31^,^39^, and Mott insulators like LaTiO_3_^38^. In this work, we have narrowed the materials search space by screening out the Mott insulators and topological insulators. This is mainly based on two reasons: (i) the inappropriate description of the electronic states of the Mott insulators in the HT first-principles calculations; and (ii) the rare number of topological insulators in the perovskite oxides. Hence, only the conventional band insulators based on the perovskite oxides are considered at this stage. Consequently, it is reasonable to use the band gap of conventional band insulators as one descriptor. Herein, we take the band gap of the mostly widely used semiconductor material silicon (1.1 eV) as a reference value, *i.e.*, the minimum value for screening desired polar perovskites. Meanwhile, considering that the first-principles standard density functional theory (DFT) calculation systematically underestimate by about 30–40% the band gap of insulators and semiconductors^40^, herein, we thus define the third descriptor for screening a new round of candidates. That is, the calculated band gap of bulk perovskite must be larger than about 0.7 eV that is the calculated band gap value of face-centered cubic silicon within standard DFT. In addition, to improve the accuracy of the HT screening approach, we have further employed hybrid functional theory calculations within Heyd-Scuseria-Ernzerhof (HSE) formalism^41^ to yield more accurate band gaps for the selected candidate polar perovskites in the last two rounds of screening. As discussed below, these HSE-calculated band gaps will also be used for determining the band alignment between the STO substrate and polar perovskite films.

**Figure 1:**
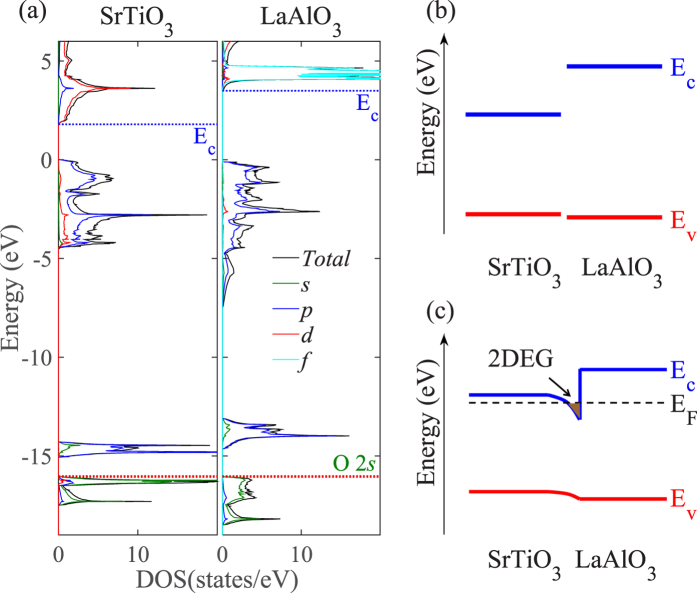
Determination of band alignment. (**a**) Calculated density of states (DOS) of bulk SrTiO_3_ (STO) and LaAlO_3_ (LAO), (**b**) band alignment of bulk LAO and STO, and (**c**) schematic band diagram for forming the 2DEG at the interface between LAO and STO. The DOS of LAO and STO was aligned according to the O 2*s* energy level.

Lastly, to form 2DEG at the interface between two oxides, another necessary condition requires that the conduction band minimum (CBM) of the electron-donor oxide (herein refers to polar perovskite which donates electrons) must be higher than that of the electron-acceptor semiconductor, *i.e.*, STO, so that the electron transfer driven by the polar catastrophe can be accumulated near the CBM, forming *n*-type conductivity. Hence, this condition can be used as the the fourth descriptor. To determine the relative band edges between two materials, one way is to reference the valence band edges of the two materials to their core level energy, and also obtain the relative positions of their core levels in an interface structure, and then to get their valence band alignment and further their conduction band alignment^42^. In this work, since the substrate and film perovskite both contain O ions, we propose to calculate the relative band alignment between the substrate oxide and candidate film oxides by aligning their core energy levels of O 2*s* orbitals. This is because O 2*s* orbital lies in the deep energy range from −20 to −15 eV relative to the valence band maximum (VBM), far away from the bonding states (valence band states) and less influenced by the atom bonding, and thus it can be used as the energy reference to an approximation. It is noted that the lattice-mismatch-induced strain and the structural distortion at the interface could alter the results to some extent, and a more accurate band alignment can be obtained using superlattice approach that, however, is not appropriate to be implemented in the HT approach. As a proof of principle, we take the prototype LAO/STO system as an example and calculate its band alignment, shown in Fig. 1. In fact, only the relative conduction band edges are used to justify the electron donor and acceptor, and the calculated relative conduction band edges are well consistent with previous results using supercell approach^1^. It is worth mentioning that these four descriptors are all based on the bulk materials properties, which do not take into account the structural relaxation effects in the HS model. As detailed below, upon structural relaxation, the strain-induced polarization in some polar oxide films can significantly influence the interfacial electronic properties, and even neutralize the polar catastrophe at the interface, prohibiting the 2DEG formation. Nevertheless, the four descriptors are still indispensable parameters to rapidly locate the target 2DEG systems before verifying the interfacial metallic states using time-consuming superlattice calculations.

**Figure 2:**
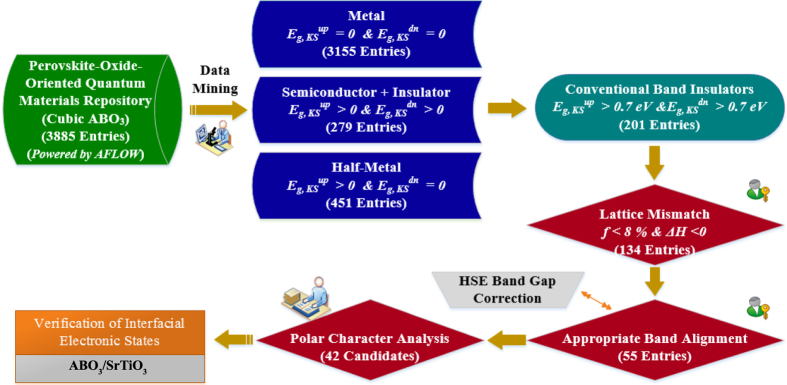
Flowchart of high-throughput screening of candidate perovskite oxides for forming two-dimensional electron gas based on polar/nonpolar heterostructures. The number of candidate perovskite oxides after each step’s screening, *i.e.*, entries for the next-step screening, is given.

**Figure 3:**
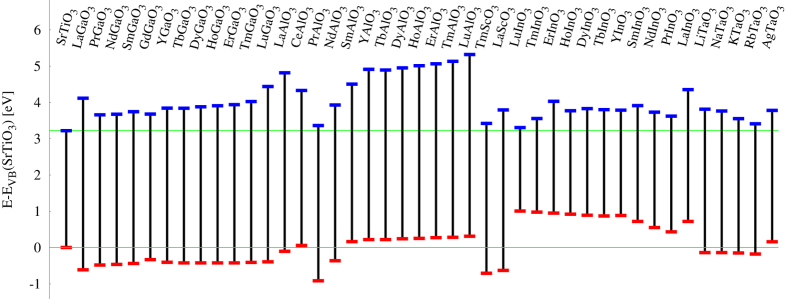
Calculated band alignment diagram between the STO substrate and candidate polar perovskite oxides.

By employing the automatic framework AFLOW^34^, we have reproduced a perovskite-oriented quantum materials database via large-scale first-principles electronic structure calculations in a HT fashion. Then by using these combinatorial descriptors, we have developed an efficient data-mining algorithm for the HT screening of perovskite-oriented quantum materials repository. The flowchart of the HT screening procedure of the candidate perovskite oxides is shown in Fig. 2, along with the number of candidate perovskite oxides after each step’s screening (or the entries for the next-step screening). After the HT screening, we rapidly identified 42 candidate systems that can potentially exhibit interfacial conducting states by forming a HS with the STO substrate. These systems are listed in Table 1, with their experimental and DFT equilibrium lattice constant *a* and lattice mismatch *f* with the STO substrate, calculated band gaps from standard DFT (GGA) and hybrid functional (HSE) approach, the relative VBM and CBM positions, and formation enthalpy. All these candidate perovskite oxides have the appropriate materials properties as defined in the descriptors: polar character, small lattice mismatch with STO, appropriate band gaps, and band edge positions. As a proof of concept, we plot the band alignment of these candidate perovskite oxides with respect to the STO substrate in Fig. 3. The calculated CBM of these perovskite oxides are all higher than that of the STO substrate, allowing charge accumulation near the CBM of the STO substrate, forming the 2DEG. These polar perovskite oxides can be subdivided into five groups of materials: Al-, Ga-, Sc-, In-, and Ta-based oxides. The Al-, Ga-, and Sc-based oxides have significantly larger band gaps than that of STO, and their CBMs are all higher than that of the STO substrate. The fourth and fifth groups of materials, In- and Ta-based oxides, have smaller band gaps than those of the former three groups of oxides, while their CBM positions are higher than that of the STO substrate. Therefore, the electronic structure characteristics of these polar oxides guarantee the appropriate band gaps and band alignments with the STO substrate for forming the 2DEG at the interfaces of HS.

**Figure 4:**
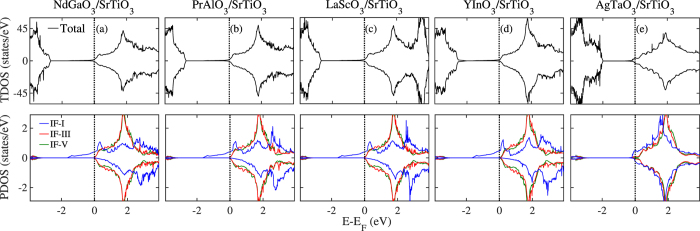
Calculated total DOS and layer-resolved partial DOS projected on Ti 3*d* orbitals for (**a**) NdGaO_3_/STO, (**b**) PrAlO_3_/STO, (**c**) LaScO_3_/STO, (**d**) YInO_3_/STO, and (**e**) AgTaO_3_/STO without structural relaxation. In this and subsequent DOS figures, the Fermi level is indicated by the vertical dash line at 0 eV.

**Figure 5:**
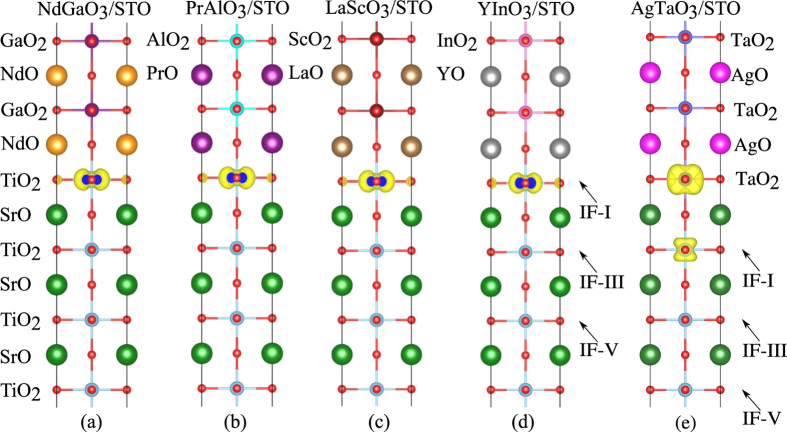
Charge density projected on the bands forming the 2DEG in the (**a**) NdGaO_3_/STO, (**b**) PrAlO_3_/STO, (**c**) LaScO_3_/STO, (**d**) YInO_3_/STO, and (**e**) AgTaO_3_/STO HS system without structural relaxation.

Next, by building HS models using these candidate perovskite oxides with STO substrate, we have calculated their electronic structures to examine whether these HS models can produce 2DEG at interface. Here we select one representative oxide from each group as a model to show its interfacial electronic property. The five selected systems are PrAlO_3_/STO, NdGaO_3_/STO, LaScO_3_/STO, YInO_3_/STO, and AgTaO_3_/STO. As mentioned above, our materials descriptors are all based on their bulk materials properties, which do not include the structural relaxation effects. Therefore, to demonstrate the rationality of our descriptors, we first calculated electronic structures of the four HS models without structural relaxation. Their density of states (DOS) plots are shown in Fig. 4. It is well known that the interfacial metallic states of the STO-based 2DEG system mainly come from the partially occupied Ti 3*d* orbitals from the interfacial TiO_2_ layer^24^,^43^,^44^. Hence, here we also calculated the partial DOS of Ti 3*d* orbitals from different TiO_2_ layers, *i.e.*, the 1st, the 3rd, and 5th layer of STO. The three TiO_2_ layers are labeled as IF-I, IF-III, and IF-V, and their partial DOS are shown in blue, red, and green, respectively. As shown in Fig. 4, the former four unrelaxed HS systems all form *n*-type half-metallic conducting states. The interfacial Ti ions from the IF-I TiO_2_ layer of the STO substrate solely contribute to the half-metallic states while deep IF-III and IF-V layers have no contribution, implying the formation of a perfectly confined 2DEG at the interface. The fifth HS system, AgTaO_3_/STO, also exhibits metallic states that is partially contributed by the IF-I TiO_2_ layer, but relatively weak spin-polarization as compared to former four HS models. Meanwhile, to have a direct view of the 2DEG spatial distribution, we also calculated three-dimensional charge density projected on the bands forming the 2DEG for these unrelaxed HS models, shown in Fig. 5. It clearly shows the 2DEG is tightly confined at the interfacial IF-I TiO_2_ layer for the former four HS systems. For the AgTaO_3_/STO, unlike the former four HS models, its interface is composed of (TaO_2_)^+^ and (SrO)^0^ layers, and the interfacial metallic states occur at interfacial TaO_2_ and IF-I TiO_2_ layers (see Fig. 5e). This indicates that the polar catastrophe at the (TaO_2_)^+^/(SrO)^0^ interface only drives part of electrons to the TiO_2_ layer, and the remaining electrons resides in the interfacial TaO_2_ layer, indicating a different electron reconstruction phenomenon from the prototypical (LaO)^+^/(TiO_2_)^0^ interface model in the LAO/STO system.

**Figure 6:**
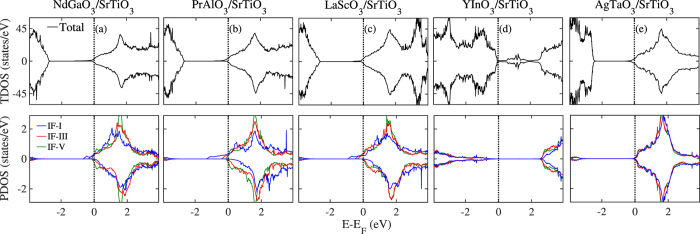
Calculated total DOS and layer-resolved partial DOS projected on Ti 3*d* orbitals for (**a**) NdGaO_3_/STO, (**b**) PrAlO_3_/STO, (**c**) LaScO_3_/STO, (**d**) YInO_3_/STO, and (**e**) AgTaO_3_/STO with structural relaxation.

**Figure 7:**
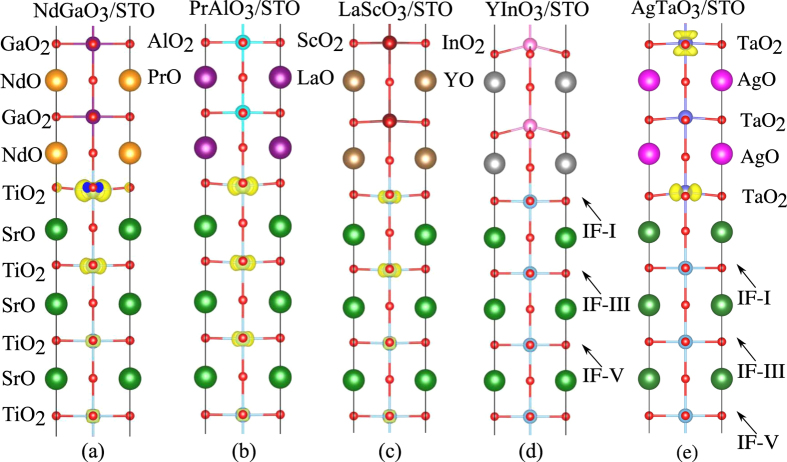
Charge density projected on the bands forming the 2DEG in the (**a**) NdGaO_3_/STO, (**b**) PrAlO_3_/STO, (**c**) LaScO_3_/STO, (**d**) YInO_3_/STO, and (**e**) AgTaO_3_/STO HS system with structural relaxation.

To further examine the influence of the lattice-mismatch-induced structural distortions on the interfacial electronic property, we have carried out the structural relaxation calculations for these HS models by minimizing their atomic forces. Their calculated total and layer-resolved DOS plots are shown in Fig. 6, along with the three-dimensional charge density forming the 2DEG in Fig. 7. For Ga-, Al-, and Sc-based oxide HS models, these systems retain the electronic structure characteristics of the 2DEG, though the 2DEG now extends to deeper TiO_2_ layers of the STO substrate, see Fig. 7. In other words, apart from the major contribution of the interfacial IF-I TiO_2_ layer, the IF-III and IF-V TiO_2_ layers also contribute to the formation of the interfacial metallic states, though their contributions are relatively small. Surprisingly, the YInO_3_/STO HS model, upon structural relaxation, does not show *n*-type conducting character, and its Fermi level is pinned just above the VBM. The layer resolved partial DOS and projected charge density plot also confirm that there are no metallic states on the interfacial TiO_2_ layer. Further analysis shows that the disappearance of the 2DEG is attributed to a strong polarization in the YInO_3_ film, which is induced by the lattice mismatch between YInO_3_ film and STO substrate, see Fig. 7d. This polarization points to the interior YInO_3_ bulk (opposite to the STO substrate), and thereby neutralizes the polar catastrophe, resulting in the dissipation of the 2DEG. For the AgTaO_3_/STO HS model, after structural relaxation, the AgTaO_3_ film exhibits a relatively weak polarization as compared to the YInO_3_/STO model (see Fig. 7e), preventing the charge transfer from TaO_2_ to TiO_2_ layer, and thus the donor electrons are confined in the AgTaO_3_ film. It is important to note that although there is no 2DEG at the interface of the YInO_3_/STO HS system, this result is still interesting because it implies that the YInO_3_ compound can be piezoelectric under compressive strain, which can lead to potentially novel functionality. Hence, it is worthy of further exploration to apply polarization effects to produce novel interfacial properties. In fact, our recent first-principles calculations on the nonpolar/nonpolar CaZrO_3_/STO HS system indicate that a strong polarization in the CaZrO_3_ film induced by a compressive strain is also capable of producing a 2DEG at the interface^45^. The different interfacial electronic property between the CaZrO_3_/STO and YInO_3_/STO systems is because of different polarization direction with respect to the STO substrate, in addition to the different polar character between the CaZrO_3_ and YInO_3_. In the former case, the polarization points to the STO substrate, which drives the charge transfer from the CaZrO_3_ to the STO substrate, forming a 2DEG; in the later case, on the contrary, the polarization points away from the STO substrate, neutralizing the polar catastrophe and inhibiting the 2DEG formation.

## Summary

In summary, we have proposed a group of combinatorial descriptors for the HT design of 2DEG systems based on perovskite oxide band insulators. These materials descriptors are based on the bulk material properties of the component film and substrate in the HS model, which include the perovskite oxides’ polar character, lattice mismatch, band gap, and band alignment with respect to the STO substrate. With this method, we have identified 42 candidate polar perovskites (some of them are already known). These candidate perovskites include the Ga-based {LaGaO_3_, PrGaO_3_, NdGaO_3_, SmGaO_3_, GdGaO_3_, YGaO_3_, TbGaO_3_, DyGaO_3_, HoGaO_3_, ErGaO_3_, TmGaO_3_, and LuGaO_3_}, Al-based {LaAlO_3_, CeAlO_3_, PrAlO_3_, NdAlO_3_, SmAlO_3_, YAlO_3_, TbAlO_3_, DyAlO_3_, HoAlO_3_, ErAlO_3_, TmAlO_3_, and LuAlO_3_}, Sc-based {TmScO_3_ and LaScO_3_}, In-based {LuInO_3_, TmInO_3_, ErInO_3_, HoInO_3_, DyInO_3_, TbInO_3_, YInO_3_, SmInO_3_, NdInO_3_, PrInO_3_, LaInO_3_}, and Ag-based {LiTaO_3_, NaTaO_3_, KTaO_3_, RbTaO_3_, and AgTaO_3_} compounds. By building HS models using these candidate perovskites with the STO substrate, and by carrying out further calculations, we have predicted that these Al-, Ga-, Sc-, and Ta-based compounds can form 2DEG with the STO substrate, while In-based compounds cannot. This is because In-based compounds exhibit strong strain-induced polarization in the HS model upon structural relaxation, which neutralizes the polar catastrophe and prohibits the 2DEG formation. It is important to note that our materials descriptors are solely based on the bulk material properties of the component film and substrate rather than a supercell-based superlattice, which ensures the practicality of the HT search for the target bulk materials and makes possible the design of perovskite-oxide-based 2DEG systems in a HT fashion.

## Methods

The automatic framework AFLOW^34^, based on the Vienna *Ab-initio* Simulation Package^46^, along with the projector augmented wave potentials^47^, and Perdew-Burke-Ernzerhof generalized gradient approximation^48^, is used to produce the quantum materials repository of the bulk perovskite oxides within the standard DFT calculations. For selected candidate perovskite oxides, the hybrid DFT calculations within Heyd-Scuseria-Ernzerhof (HSE) formalism^41^ with 25% Hartree-Fock exchange are employed to improve the band gap prediction and to determine relative conduction band edge positions between the STO substrate and polar perovskite oxide films. In the modeling of STO-based HS, considering that strong correlation effects of Ti 3*d* electrons, the spin-polarized generalized gradient approximation (GGA) plus on-site Coulomb interaction approach (GGA+*U*) is applied in the electronic structure calculation of the HS models with *U *= 5.8 eV for the Ti 3*d* orbitals^49^,^50^. A supercell approach is used to build the HS models in which the film and substrate is modeled using 6 and 11 unit cells, respectively, and the DFT equilibrium lattice constant 3.945 Å of STO is used. For NdGaO_3_/STO, PrAlO_3_/STO, LaScO_3_/STO, and YInO_3_/STO HS models, symmetrical *n*-type (*A*O)^+1^/(TiO_2_)^0^ (*A* = Nd, Pr, La, and Y) interfaces are modeled. For AgTaO_3_/STO HS model, a symmetrical *n*-type (TaO_2_)^+^/(SrO)^0^ interface is modeled. A kinetic energy cutoff of 450 eV is used for the electronic wave function expansion. A 10 × 10 × 1 *k*-space grid is used for the HS calculations. All the crystal structures are optimized by minimizing the atomic forces smaller than 0.02 eV/Å. Self-consistency is assumed for a total energy convergence of less than 10^−5^ eV.

## Additional Information

**How to cite this article**: Yang, K. *et al*. High-Throughput Design of Two-Dimensional Electron Gas Systems Based on Polar/Nonpolar Perovskite Oxide Heterostructures. *Sci. Rep.* **6**, 34667; doi: 10.1038/srep34667 (2016).

## Figures and Tables

**Table 1:** Properties of bulk perovskite oxides: experimental and DFT equilibrium lattice constant *a* (Å) and lattice mismatch *f* with the STO substrate, calculated band gaps *E*_*g*_ (eV) from GGA and HSE approaches, valence band maximum (VBM) and conduction band minimum (CBM) positions, and formation enthalpy (eV/atom).

Compound	Exp. *a*(Å)	Exp. *f* (%)	DFT *a* (Å)	DFT *f* (%)	DFT(GGA)	DFT(HSE)	VBM(eV)	CBM(eV)	ΔH(eV)
					*E*_*g*_ (eV)	*E*_*g*_(eV)			
SrTiO_3_	3.905^8^	0	3.945	0	1.79	3.22	0	3.22	−3.22
*LaGaO_3_	3.860^51^	−1.15	3.929	−0.41	3.34	4.73	−0.62	4.11	−2.66
PrGaO_3_	—	—	3.913	−0.81	2.77	4.14	−0.48	3.66	−2.59
*NdGaO_3_	3.846^22^	−1.51	3.901	−1.12	2.75	4.14	−0.47	3.67	−2.56
SmGaO_3_	3.850^52^	−1.41	3.883	−1.57	2.75	4.18	−0.44	3.74	−2.54
*GdGaO_3_	3.840^52^	−1.66	3.876	−1.75	1.78	4.01	−0.33	3.68	−2.48
YGaO_3_	—	—	3.862	−2.10	2.67	4.25	−0.41	3.84	−2.45
TbGaO_3_	3.833^52^	−1.84	3.860	−2.15	2.80	4.26	−0.42	3.84	−2.49
DyGaO_3_	3.825^52^	−2.05	3.855	−2.28	2.82	4.30	−0.42	3.88	−2.47
HoGaO_3_	3.813^52^	−2.36	3.850	−2.41	2.85	4.33	−0.42	3.91	−2.45
ErGaO_3_	3.808^52^	−2.48	3.845	−2.54	2.88	4.36	−0.42	3.94	−2.43
TmGaO_3_	3.798^52^	−2.74	3.839	−2.69	2.93	4.43	−0.41	4.02	−2.40
LuGaO_3_	3.782^52^	−3.15	3.832	−2.86	2.99	4.83	−0.39	4.44	−2.36
*LaAlO_3_	3.789^8^	−2.97	3.811	−3.40	3.49	4.92	−0.11	4.81	−3.33
CeAlO_3_	3.773^53^	−3.38	3.810	−3.42	2.91	4.27	+0.06	4.32	− 3.24
*PrAlO_3_	3.772^22^	−3.41	3.790	−3.93	2.88	4.28	−0.92	3.36	−3.27
*NdAlO_3_	3.764^22^	−3.61	3.774	−4.33	2.87	4.29	−0.36	3.93	−3.26
SmAlO_3_	3.738^53^	—	3.749	−4.97	2.87	4.34	+0.16	4.50	−3.26
YAlO_3_	—	—	3.718	−5.75	2.80	4.69	+0.22	4.91	−3.19
TbAlO_3_	3.718^53^	−4.79	3.717	−5.78	2.93	4.67	+0.22	4.89	−3.22
DyAlO_3_	3.713^53^	−4.92	3.709	−5.98	2.95	4.71	+0.24	4.95	−3.21
HoAlO_3_	3.704^53^	−5.15	3.702	−6.16	2.98	4.76	+0.25	5.01	−3.20
ErAlO_3_	3.698^53^	−5.30	3.695	−6.34	3.01	4.79	+0.27	5.06	−3.19
TmAlO_3_	3.688^53^	−5.56	3.687	−6.54	3.05	4.85	+0.28	5.13	−3.17
LuAlO_3_	3.676^53^	−5.86	3.677	−6.79	3.13	5.01	+0.31	5.32	−3.14
TmScO_3_	3.916^54^	+0.28	4.000	−1.39	2.57	4.13	−0.71	3.42	−2.99
LaScO_3_	4.053^55^	+3.79	4.076	+3.32	2.82	4.42	−0.80	3.62	−3.37
LuInO_3_	—	—	4.108	+4.13	0.77	2.30	+1.00	3.30	−1.81
TmInO_3_	—	—	4.112	+4.23	1.06	2.58	+0.97	3.55	−1.86
ErInO_3_	—	—	4.116	+4.33	1.24	3.08	+0.95	4.03	−1.90
HoInO_3_	—	—	4.119	+4.41	1.39	2.85	+0.92	3.77	−1.93
DyInO_3_	—	—	4.123	+4.51	1.54	2.94	+0.89	3.83	−1.96
TbInO_3_	—	—	4.126	+4.59	1.62	2.93	+0.87	3.80	−1.98
YInO_3_	—	—	4.129	+4.66	1.50	2.90	+0.88	3.78	−1.95
SmInO_3_	—	—	4.142	+4.99	1.62	3.19	+0.72	3.91	−2.01
NdInO_3_	—	—	4.155	+5.32	1.65	3.18	+0.55	3.73	−2.06
PrInO_3_	—	—	4.163	+5.53	1.69	3.19	+0.43	3.62	−2.09
LaInO_3_	—	—	4.175	+4.83	2.04	3.63	+0.72	4.35	−2.21
LiTaO_3_	—	—	3.963	+0.46	2.32	3.95	−0.14	3.81	−2.58
NaTaO_3_	3.931^56^	+0.67	3.981	+0.91	2.27	3.90	−0.14	3.76	−2.65
KTaO_3_	3.989^57^	+2.15	4.030	+2.12	2.10	3.70	−0.15	3.55	−2.65
RbTaO_3_	4.029^58^	+3.18	4.071	+3.19	2.01	3.59	−0.18	3.41	−2.57
AgTaO_3_	3.950^59^	+1.15	3.999	+1.37	1.73	3.62	+0.16	3.78	−1.99

^*^Indicates that a 2DEG has been experimentally validated.
